# Development of a scale measuring home-visiting nurses’ attitudes toward patient safety: a cross-sectional study

**DOI:** 10.1186/s12912-023-01288-8

**Published:** 2023-05-06

**Authors:** Keiko Yoshimatsu, Hisae Nakatani

**Affiliations:** 1 Department of Nursing, Faculty of Nursing and Nutrition, The University of Shimane, 151 Nisihayasigi-cho, Izumo-shi, Shimane-ken, 693-8550 Japan; 2 Department of Community and Public Health Nursing, Graduate School of Biomedical and Health Sciences, Hiroshima University, 1-2-3 Kasumi, Minami-ku, Hiroshima City, Hiroshima, 734-8551 Japan

**Keywords:** Home-visiting nurses, Patient safety, Attitude scale

## Abstract

**Background:**

Home-visiting nurses are required to recognize risks in their work, ensure patient safety according to the characteristics of home-visiting nursing, and therefore, effectively support stability in patients’ lives. In this study, we created a scale measuring home-visiting nurses’ attitudes toward patient safety and examined its reliability and validity.

**Methods:**

A total of 2,208 home-visiting nurses from Japan were randomly selected as participants. From the 490 responses collected (response rate: 22.2%), 421 responses with no missing values, other than those related to participants’ basic information (valid response rate: 19.0%), were analyzed. Participants were randomly divided into two groups: 210 for exploratory factor analysis (EFA) and 211 for confirmatory factor analysis (CFA). To examine the reliability of the home-visiting nurses attitude scale developed in this study, ceiling and floor effects, inter-item correlations, and item-total correlations were checked. Subsequently, EFA was performed to confirm the factor structure. CFA, composite reliability, average variance extracted, and Cronbach’s alpha for each factor were extracted to confirm the factor structure of the scale and the validity of the model.

**Results:**

The home-visiting nurses’ attitudes toward patient safety were measured using 19 questionnaire items related to four factors: “Self-improvement for patient safety,” “Incident awareness,” “Counter measures based on incident experience,” and “Nursing care to protect the lives of patients.” Cronbach’s α coefficients were 0.867, 0.836, 0.773, and 0.792 for Factors 1–4, respectively. Model indicators were χ^2^ = 305.155, df = 146, p < 0.001, TLI = 0.886, CFI = 0.902, RMSEA = 0.072 (90% confidence interval 0.061–0.083).

**Conclusions:**

From the results of the CFA, criterion-related validity, and Cronbach’s α coefficient, this scale is considered reliable and valid and thus, highly appropriate. Therefore, it may be effective at measuring home-visiting nurses’ attitudes toward patients’ medical safety from both behavioral and awareness aspects.

## Background

The Japanese population is aging rapidly, and the number of patients requiring long-term care and medical care is expected to increase. As a national policy, the establishment of the community-based integrated care system is being promoted so that those needing care can continue to live in a familiar area [1]. Unlike hospital-based care, home care does not always mean having a medical professional nearby; rather, those needing care typically spend more time with family alone. Therefore, family caregivers provide domestic and medical care and are required to respond to problems. A home-visiting nurse’s (HVN’s) role is to support home-based patients in need of medical care.

HVNs must strive to anticipate and reduce possible risks at the patient’s home for the patient to continue to live safely. They must ensure this by coordinating with various professionals and assist patients and their families deal with potential risks. Another important aspect of ensuring patient safety is consulting information from incident reports related HVNs to and home care. However, one study [2] found that among the 70% HVNs who experienced incidents, 40% did not report their incidents. Further, notably, most incidents that occur in the absence of HVNs are not reported [3]. Information regarding such incidents is believed to be obtained from patients and their families. Although patient-reported incidents are important for preventing recurrence, such reports by patients rarely lead to corrective action in the actual system [4]. Nevertheless, knowing how HVNs share, consider, and use information obtained from patients and their families may be important to prevent risks and improve the system of home-visiting nursing agencies. Additionally, as HVNs often visit patients’ homes alone, they are rarely monitored by others. Therefore, it is presumed that there may be a shortage of incident reports and safety discussions due to individual factors such as the HVNs’ perception of patient safety.

Home-visiting nursing agencies are considered to address patient safety through administrators systematically. However, administrators have more incident experiences and higher occupational stress than staff HVNs [5]. In Japan, in addition to administrative duties, administrators also visit patients at home. Owing to heavy workload, administrators may limit patient safety efforts, such as hosting training sessions and thorough incident reporting. Consequently, it is important for individual HVNs to actively participate in the safety efforts of home-visiting nursing agencies by improving patient safety awareness and attitudes at the individual level.

Attitudes are composed of three components: cognition, emotions, and behavior [6], and nurse attitudes are sometimes used to assess patient safety [7, 8]. It may be useful to measure attitudes toward patient safety to evaluate individual HVNs’ efforts to ensure the same. Behavioral measures assessing safety culture in hospital nursing units [9] and nurses’ attitudes and skills toward patient safety [10] have been developed. These are indicative of nurses’ safety behaviors and attitudes in the hospital environment. However, the unique characteristics of home care can make it difficult to use or modify effective safety interventions developed for other situations. Therefore, research on effective practices implemented in a home care environment is needed [11]. As an attitude toward risk management, HVNs need to tailor care to patients and their families and work with them to prevent adverse events [12]. To support the stability of a patient’s life, HVNs must be aware of the risks in patients’ daily lives and safeguard patient safety according to the characteristics of home-visiting nursing. Thus, it is necessary to develop a scale measuring HVNs’ attitudes toward patient safety based on the relevant occupational characteristics. This study was developed based on a previous interview survey conducted by the researchers [12] that clarified HVN’s attitudes toward patient safety from three perspectives: cognitive, emotional, and behavioral. Through such a scale, it may be possible to encourage HVNs to reflect on their own behavior and actively perform their duties, which could improve patients’ safety. Hence, this study develops an HVN attitude scale based on patient safety and examines its reliability and validity.

## Methods

### Participants

In Japan, a full-time equivalent (FTE) of 2.5 or more is advised when staffing home-visiting nursing agencies with public health nurses, midwives, registered nurses, or licensed practical nurses [13]. The average FTE per HVN establishment is 5.0 [14]. As of 2021, there are 13,444 home-visit nursing stations, with 133,845 employees, of which 61.0% are registered nurses [15]. This study’s participants were HVNs working at visiting nurse stations across Japan. Using the Ministry of Health, Labour and Welfare’s Nursing Care Service Information Disclosure System, home-visiting nursing agencies were extracted. Different HVNs were used for exploratory factor analysis (EFA) and confirmatory factor analysis (CFA). EFA requires at least five participants per item and CFA requires a minimum of 200 participants [16, 17]. Therefore, a total of 375 HVNs were required, with a minimum of 175 for EFA and 200 for CFA. The questionnaires were distributed by mail with the aim of collecting data from 400 HVNs, 200 for each analysis. Home-visiting nursing agencies were divided into three groups based on size: 2.5 to 5 FTEs, 5 to 7 FTEs, and 7 or more FTEs. Home-visiting nursing agencies were selected using a random number table so that the number of target HVNs would be the same for each size group. There were approximately 735 HVNs in each group, and a total of 2,208 HVNs were included. We distributed 2,208 copies of the questionnaire and received responses from 490 people (response rate: 22.2%). After excluding those with missing data in parts other than that related to the participants’ characteristics, a total of 421 responses from the nurses were included in the analysis (valid response rate: 19.1%). Participants were randomly divided into two groups for EFA (n = 210) and CFA (n = 211) analysis.

### Creation of items

The items pertaining to the HVNs’ attitudes toward patient safety were created in reference to the content of an interview survey the researchers had conducted in advance [12]. For the initial items, we sought the opinions of university faculty members and graduate students majoring in community nursing and made revisions to determine whether they showed attitudes toward patient safety. The initial number of items was 35. These items demonstrate HVNs’ cognition, emotions, and behavior toward patient safety adopted in their nursing practice. To determine whether the questionnaire items adequately capture HVNs’ attitudes toward patient safety, they were validated by 10 HVNs who have been practicing in home-visiting nursing care for more than 10 years; content validity was also examined. The 10 HVNs were nurses who not only had home-visit nursing experience but also nursing experience in various departments, such as hospital wards and outpatients. Specifically, they focused on determining whether the questionnaire items (1) were correctly expressed, (2) reflected the HVNs’ attitudes toward patient safety, (3) adequately captured such attitudes, and (4) presented any bias in their content. It was also examined whether item content was related to HVNs’ attitudes toward patient safety. Items’ content validity index (CVI) value must be at least 0.8 [16]. Questionnaire items for which more than 80% of the HVNs responded that the content was related to patient safety attitudes were selected. Based on the validity findings, the questionnaire was revised, and all 35 items were retained.

### Data collection

The questionnaire was distributed to each institution by mail. Subjects were requested to complete an anonymous, self-administered questionnaire. Upon completion, the questionnaires were sealed and mailed to the researcher. The data collection period was from November 2021 to February 2022.

### Measures

The survey included HVNs’ basic information, items on their attitudes toward patient safety, and two criterion tools.

#### Basic information

The basic information included age, years of nursing experience, years of home-visiting nursing experience, employment pattern, job title, qualifications, and whether they go on night standby. Based on the characteristics of HVNs’ workplaces, we divided them into three groups according to the number of FTEs at home-visit nursing institutions. In Japan, full-time hours are determined by the workplace. Generally, the FTE calculation is 32–40 h per full-time employee per week [13, 14].

#### HVNs’ attitudes toward patient safety

HVNs’ attitudes toward patient safety were rated on a 4-point Likert scale, ranging from 1 (never) to 4 (all of the time).

#### Criterion-related validity

The following was used as a measure of co-existing criterion-related validity with the permission of the creators: the tool that measures the workplace safety climate among hospital female nurses in Japan [18] (hereafter, the workplace safety climate measure) and the revised edition of the nurse’s ethical behavior scale [19] (hereafter, the revised ethical behavior scale). In home care, organizational learning is recognized as the most positive safety culture, and knowledge of safety culture can lead to systematic improvement and avoidance of patient safety accidents [20]. Based on the idea that the safety culture of the business establishments where they work affects HVNs, a study investigated workplace safety climate and found that the quality of ethics in nursing affects that of nursing and patient safety [21]. Attitudes toward patient safety are assumed to be largely related to the judgments and ethics of individual nurses. Therefore, the relationship between HVNs’ attitudes toward patient safety and their ethical behaviors was examined. The workplace safety climate measure was developed by Sakita and verified for reliability; the Cronbach’s alpha for the overall scale was 0.74 in the original study [18]. There are 20 items, and they are answered on a 5-point scale ranging from “1 = Strongly disagree” to “5 = Strongly agree”; the higher the score, the better the workplace safety organizational culture [18]. The revised ethical behavior scale was developed by Ode and verified for reliability; Cronbach’s alpha was 0.78 for “risk avoidance” (5 items), 0.75 for “good care” (5 items), 0.74 for “fair care” (5 items), and 0.84 for the total scale [19]. There are 15 items, and they are measured on a 6-point scale ranging from “1 = Not at all” to “6 = Very much”; the higher the score, the higher the nurse’s sense of ethics [19]. The Cronbach’s α coefficients of two scales are > 0.7 [16].

### Data analysis

IBM SPSS Statistics for Windows, version 26 (IBM Corp., Armonk, N.Y., USA) and Amos version 26 (Amos Development Corporation, 2019) were used for the analysis. Comparisons of EFA and CFA participant characteristics were performed using the chi-square test, and Fisher’s exact test for items with a cell size of 5 or less.

#### Reliability

To examine the reliability of the HVN attitude scale developed in this study, ceiling and floor effects, inter-item correlations, and item-total (I-T) correlations were checked. The ceiling and floor effects were set to mean + standard deviation above 4 points, the floor effect was set to mean – standard deviation below 1 point, and the contents were examined [16, 17]. Highly correlated coefficients may affect the results [16], so referring to previous research [22], the inter-item correlation was set at r > 0.75, and scale items were selected excluding highly important items. For I-T correlation, r < 0.2 was set as a criterion for exclusion.

Additionally, the Cronbach’s α coefficients for each factor and the overall scale were calculated. The criterion for Cronbach’s α coefficient was > 0.7 [16].

#### Exploratory factor analysis (EFA)

To confirm the factor structure, EFA (maximum likelihood method and Promax rotation) was conducted. Items with a factor loading of 0.4 or higher [16] and whose Cronbach’s α coefficient did not decrease drastically when deleted were adopted to confirm the factor structure and name the factors rationally and appropriately.

#### Confirmatory factor analysis (CFA)

CFA confirmed the validity of the scale factor structure and model. For the goodness of fit of the model, Tucker-Lewis Index (TLI), comparative fit index (CFI), and root mean square error of approximation (RMSEA) were used. The criteria for the goodness of fit of the model were TLI > 0.9, CFI > 0.9, and RMSEA < 0.08 [16]. Cronbach’s α coefficient, composite reliability (CR), and average variance extracted (AVE) were calculated to examine the reliability and convergent validity of each factor. The criteria were set at Cronbach’s alpha > 0.7, CR > 0.5, AVE > 0.7, and CR > AVE [16, 23, 24].

#### Criterion-related validity

To examine criterion-related validity, the relationship between the developed scale and the workplace safety climate measure [18] and the revised ethical behavior scale [19] was examined using Spearman’s rank correlation coefficient. No normality was found in the HVNs’ attitude toward patient safety scale and the revised ethical behavior scale; the Shapiro-Wilk normality test was performed for each.

### Ethical considerations

This study was approved by The University of Shimane Izumo Campus Research Ethics Committee (authorization number 345). Informed consent was obtained from study participants, including consent to publish the findings. All methods were conducted in accordance with the Declaration of Helsinki ethical guidelines.

## Results

### Participant characteristics

The characteristics of the participants are shown in Table 1. The average age of the participants was 47.9 ± 9.1 years, with an age range of 25–67 years; 184 (43.7%) participants were over 50 years old. The majority (184; 34.9%) had been nurses for 20 years or more but less than 30 years, with an average experience of 23.5 ± 9.4 years. The average years of experience as an HVN was 7.0 ± 6.6 years, with 108 (25.7%) respondents having over 10 years of experience as HVNs. Regarding employment patterns, 324 respondents (77.0%) were full-time and 96 (22.8%) were part-time HVNs. Further, there were 76 (18.1%) administrators and 343 (81.5%) staff HVNs. FTE was 137 (32.5%) in the ≥ 2.5, < 5 group, 146 (34.7%) in the ≥ 5, <7 group, and 138 (32.8%) in the ≥ 7 group. Moreover, 267 (63.4%) respondents were on night standby duty, while 154 (36.6%) were not. No differences were found between EFA and CFA participants.

**Table 1:** Participant characteristics

		Total(n = 421)		EFA(n = 210)		CFA(n = 211)		chi-square values	
Item		n	%	n	%	n	%		P
Age	20–39 years	84	20.0	34	16.2	50	23.7	4.131	0.124
	40–49 years	149	35.4	81	38.6	68	32.2		
	≥ 50 years	184	43.7	91	43.3	93	44.1		
	No answer	4	1.0	4	1.9	0	0.0		
Years of nursing experience	< 10 years	31	7.4	13	6.2	18	8.5	7.109	0.068
	≥ 10 years, < 20 years	118	28.0	52	24.8	66	31.3		
	≥ 20 years, < 30 years	147	34.9	86	41.0	61	28.9		
	≥ 30 years	125	29.7	59	28.1	66	31.3		
Years of experience as home-visiting nurse	< 2 years	100	23.8	49	23.3	51	24.2	0.589	0.906
	≥ 2 years, < 5 years	103	24.5	50	23.8	53	25.1		
	≥ 5 years, < 10 years	107	25.4	57	27.1	50	23.7		
	≥ 10 years	108	25.7	54	25.7	54	25.6		
	No answer	3	0.7	0	0.0	3	1.4		
Employment pattern	Full-time employment	324	77.0	159	75.7	165	78.2	0.486	0.561
	Part-time employment	96	22.8	51	24.3	45	21.3		
	No answer	1	0.2	0	0.0	1	0.5		
Job title	Administrator	76	18.1	41	19.5	35	16.6	1.482	0.526
	Staff HVN	343	81.5	169	80.5	174	82.5		
	No answer	2	0.5	0	0.0	2	0.9		
On night standby duty	Performing	267	63.4	129	61.4	138	65.4	0.717	0.419
	Not performing	154	36.6	81	38.6	73	34.6		
Full-time equivalent	≥ 2.5, < 5	137	32.5	68	32.4	69	32.7	0.061	0.977
	≥ 5, < 7	146	34.7	74	35.2	72	34.1		
	≥ 7	138	32.8	68	32.4	70	32.8		

EFA/CFA: chi-square test, items with a cell size of 5 or less are Fisher’s exact test

### Reliability

A ceiling effect was found for 18 items. As this scale is a four-point measure that is not normally distributed, items were carefully examined for content without deletion. Instead, content was analyzed with inter-item correlations, I-T correlations, and factor analysis. Conversely, no floor effect was found. The inter-item correlation showed a strong correlation of r = 0.791 (p < 0.001) between Items 29 and 30. Item 29 was “Incidents can happen to any patient,” and Item 30 was “Incidents can happen to you.” Further, Item 30 was selected as it was considered more indicative of the attitude of HVNs. I-T correlations were calculated, and Items 25, 34, and 35, which had r ≤ 0.3 [16], were deleted. Item 25 was “Even if there is a risk at the patient’s home, it is difficult to surface.” Item 34 was “I am worried that my behavior will become a habit.” Item 35 was “The perception of incidents differs for each HVN.”

### EFA

EFA was performed by maximum likelihood and promax rotation. Before EFA, the Kaiser-Meyer-Olkin (KMO) test and Bartlett’s sphericity test were performed to determine the fit of the data for factor analysis. KMO has a standard value of 0.8–1.0 and a significance probability of < 0.05 in Bartlett’s sphericity test, and it is considered valuable for factor analysis [23]. KMO was 0.883 and Bartlett’s sphericity test p < 0.001 confirmed the goodness of fit of EFA. The Gutmann criterion [23, 25] was used to extract the factors; the number of factors showing a value of 1.0 or more is the number of eigenvalues obtained from the eigen decomposition of the correlation matrix. A four-factor structure that showed 1.0 or more from the eigen analysis of the correlation matrix was adopted.

After repeated deletion of items with factor loadings of less than 0.4, considering the performance of EFA, and calculating Cronbach’s α coefficient, four factors comprising items with factor loadings of 0.4 or more [16] were finally adopted (Table 2). The following 12 items were deleted: Item 1 “Patient safety awareness,” Item 2 “Collaboration with other professionals on patient safety,” Item 3 “Prompt response to incidents,” Item 7 “Prioritize patient safety,” Item 10 “Responsibility for nursing care,” Item 14 “Concentrate on care without danger,” Item 15 “Resolve anxiety in nursing care in advance,” Item 20 “Share information with patients and families on a regular basis,” Item 21 “Nursing care according to procedures,” Item 22 “For patient safety, I provide nursing care with plenty of time,” Item 24 “Caregiving power leads to risks,” and Item 28 “Incidents lead to disadvantages for caregivers.” The four factors were: Factor 1–Self-improvement for patient safety (7 items), Factor 2–Incident awareness (4 items), Factor 3–Counter measures based on incident experience (5 items), and Factor 4–Nursing care to protect the lives of patients (3 items). The final EFA gave a KMO of 0.875 and a Bartlett’s sphericity test of 1790.254 (p < 0.001). Cronbach’s α coefficients were 0.867, 0.836, 0.773, and 0.792 for Factors 1–4, respectively. The overall Cronbach’s α coefficient was 0.885. Cronbach’s α coefficient was > 0.7 [16] for all items, confirming reliability. The correlations among the four factors ranged from r = 0.251–0.632, yielding weak to moderate correlation coefficients.

**Table 2:**
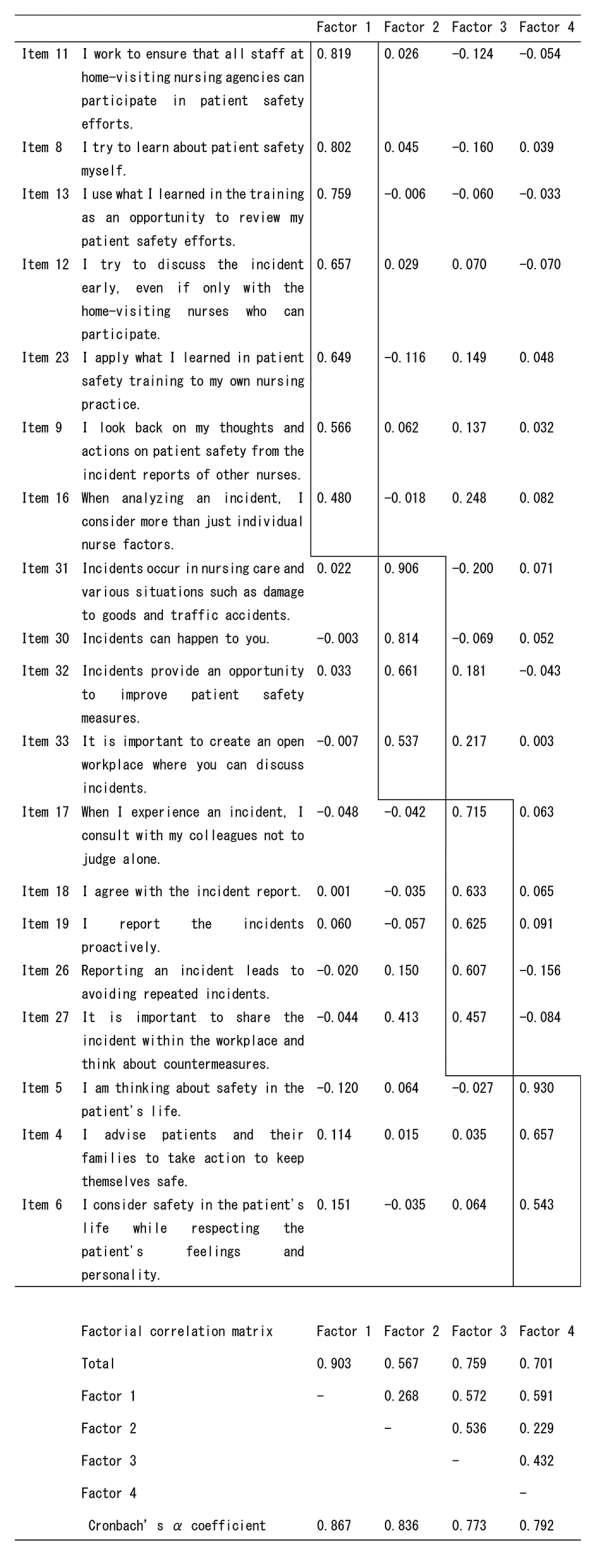
EFA of home-visiting nurses’ attitudes toward patient safety

### CFA

**Fig. 1:**
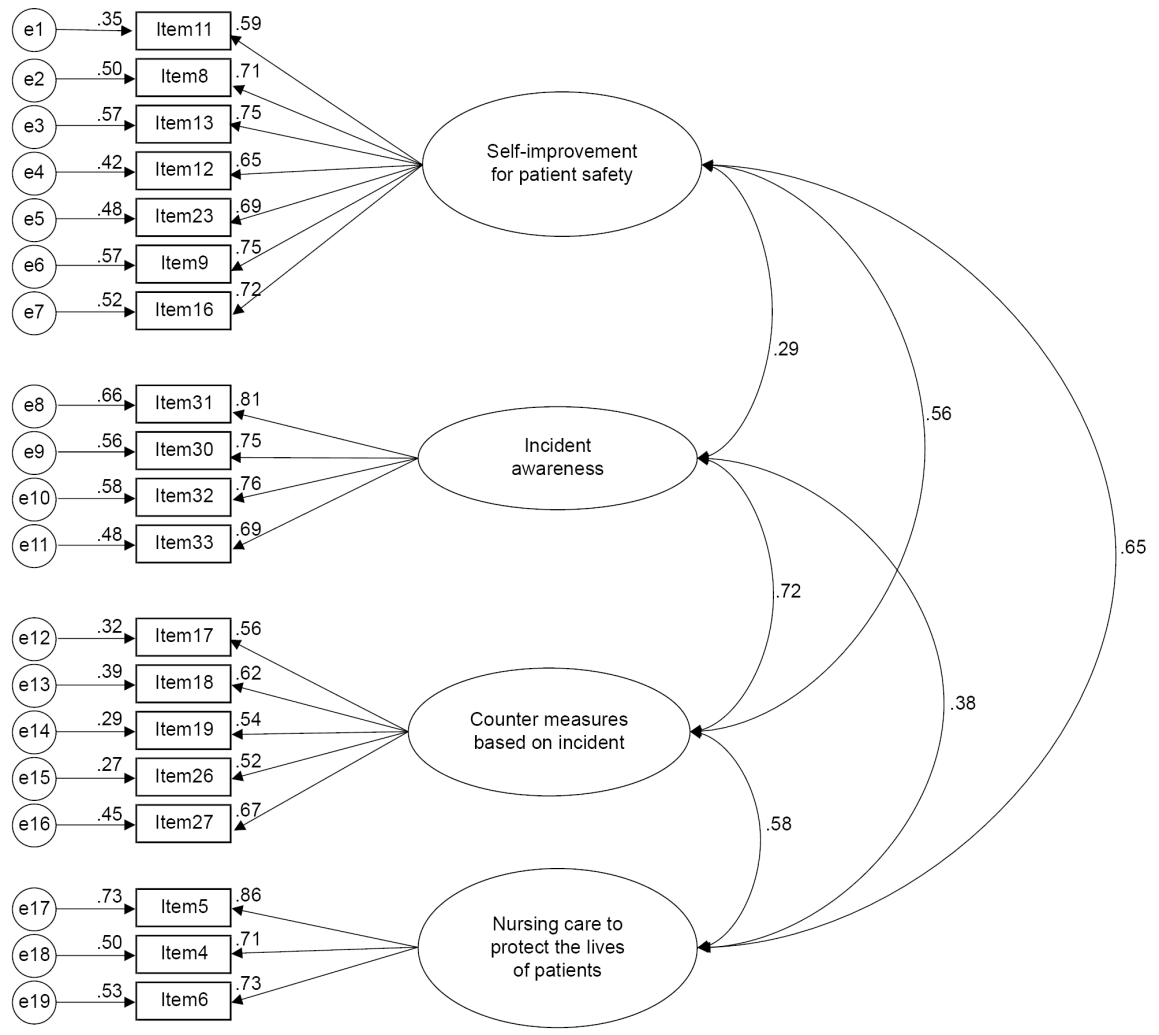
Confirmatory factor analysis of home-visiting nurses’ attitudes toward patient safety χ^2^ = 305.155, df = 146, p < 0.001, TLI = 0.886, CFI = 0.902, RMSEA = 0.072 (90% confidence interval 0.061–0.083)

CFA was performed to verify the factor structure obtained from EFA. CFA of 19 items across four factors was performed. Figure 1 shows the results of the analysis performed through the maximum likelihood estimation method. The scale was not normally distributed. Since the maximum likelihood estimator is based on the assumption of multivariate normality, we used the bootstrap method provided in Amos [26]. Model indicators were χ^2^ = 305.155, df = 146, p < 0.001, TLI = 0.886, CFI = 0.902, RMSEA = 0.072 (90% confidence interval 0.061–0.083).

Cronbach’s alpha coefficients for the four factors were 0.87, 0.84, 0.72, and 0.80, respectively. All factors were > 0.7. The AVEs of the four factors were 0.52, 0.65, 0.37, and 0.67, respectively, and CR was 0.88, 0.88, 0.73, and 0.86, respectively. All CRs were greater than AVE (Table 3). Factors 1, 2, and 4 had AVE > 0.5 and CR > 0.7. Factor 3 had an AVE of < 0.5, but a CR greater than AVE and greater than or equal to > 0.7. Even if AVE is less than 0.5, convergence validity is considered sufficient if CR is > 0.6 [16, 23]. Therefore, the convergent validity of this measure was considered acceptable.

**Table 3:** Content validity of home-visiting nurses’ attitudes toward patient safety

	Total Score		Cronbach’s α coefficient	AVE	CR
	r	P-value			
Factor 1	0.887	< 0.0001	0.87	0.52	0.88
Factor 2	0.492	< 0.0001	0.84	0.65	0.88
Factor 3	0.764	< 0.0001	0.72	0.37	0.73
Factor 4	0.707	< 0.0001	0.80	0.67	0.86

Spearman’s rank correlation coefficient between the score of four factors and the total score.

### Criterion-related validity

To examine the criterion-related validity, we determined the correlation coefficient between the HVNs’ attitude toward patient safety scale, the workplace safety climate measure, and the revised ethical behavior scale (Table 4).

**Table 4:** Criterion-related validity verification

	Overall scale	Factor 1	Factor 2	Factor 3	Factor 4
Measurement of the workplace safety climate among hospital female nurses in Japan	0.372**	0.330**	0.170*	0.322**	0.204**
Revised edition of the nurse’s ethical behavior scale: Risk aversion	0.525**	0.453**	0.239**	0.340**	0.517**
Revised edition of the nurse’s ethical behavior scale: Good care	0.594**	0.529**	0.268**	0.416**	0.523**
Revised edition of the nurse’s ethical behavior scale: Fair care	0.308**	0.246**	0.170*	0.187**	0.294**

Spearman’s rank correlation coefficient

**p < 0.001, *p < 0.01

In relation to the workplace safety climate measure, there was a weak correlation between the entire scale and all factors. The values were as follows: overall scale, r = 0.372(p < 0.001); Factor 1, r = 0.330(p < 0.001); Factor 2, r = 0.170 (p = 0.014); Factor 3, r = 0.322(p < 0.001); Factor 4, r = 0.204 (p = 0.003). There was also a correlation between the new scale and the revised ethical behavior scale. The new scale and “risk aversion” values were: overall scale, r = 0.525; Factor 1, r = 0.453; Factor 2, r = 0.239; Factor 3, r = 0.340; Factor 4, r = 0.517 (all p < 0.001). The new scale and “good care” values were: overall scale, r = 0.594; Factor 1, r = 0.529; Factor 2, r = 0.268; Factor 3, r = 0.416; Factor 4, r = 0.523 (all p < 0.001). The new scale and “fair care” values were: overall scale, r = 0.308 (p < 0.001); Factor 1, r = 0.246 (p < 0.001); Factor 2, r = 0.170 (p = 0.014); Factor 3, r = 0.187 (p = 0.006); Factor 4, r = 0.294 (p < 0.001).

## Discussion

This study demonstrated that a scale measuring HVNs’ attitudes toward patient safety has goodness-of-fit and can be used in the context of home-visiting nursing.

### Contents of the scale

The developed HVNs’ patient safety attitude scale comprised 19 items across 4 factors. These are aimed at improving nurses’ patient safety awareness and behavior by measuring the following nursing practices. First, “self-improvement for patient safety” included the nurses’ participation and reflection during training to practice ensuring patient safety. Such participation in regular error management training is necessary to improve patient safety [27]. Also included was the sharing of training content among HVNs. Many of the home-visiting nursing agencies in Japan are small [13], which can make it difficult for HVNs to participate in training. Complementary working between team members may ensure adequate competence levels [28]. For patient safety, it is necessary to consider not only the quality of one’s own nursing care but also one’s attitude as a team member. The more developed the agency’s safety culture, the less likely nurses are to fail at providing care [29]. This factor includes items such as nurses taking responsibility for the nursing practice as well as assessing patient risk and preventing medical accidents.

Second, “incident awareness” included the ability to anticipate that incidents could occur at any time. Patients and family caregivers prefer home care, despite safety concerns [30]; as such care entails nurses visiting alone, the nurses should have the flexibility to make decisions on the spot. Restrictions such as those imposed on the time at which the next patient’s home visit is scheduled and the contracted time often cause a feeling of time tension. Additionally, adverse events in home care are more frequent among agencies that have many patients with high care needs [31]. As patients become more dependent on medical care, it is presumed that in addition to providing medical care, the content of guidance provided to family members, such as how to provide care and how to deal with problems, will increase. Therefore, HVNs are required to provide various nursing care services within a set period. While doing this, it is important not to overlook any form of care provision. It is important for HVNs to have an attitude of always being aware of incidents so as not to miss any risks for patient safety.

Third, “counter measures based on incident experience” includes nurse behavior regarding incident reporting and participation in reporting and discussion. The World Health Organization states that learning from adverse events can contribute to ensuring patient safety [32]. In addition, the International Council of Nurses indicates that nurses are committed and accountable for patient safety, including improving patient safety through risk reduction, adverse event reporting, education, and research [33]. It is important to report the incident and use the experience to improve the system of the home-visiting nursing agencies. Nurses who are afraid of being attached to or accused of wrongdoing do not always report adverse events; thus, it is important to cultivate a positive safety culture [34]. Therefore, the new scale includes items related to information sharing and creating a work environment where countermeasures can be considered. It is important to not only increase self-improvement by reflecting on one’s own incident experience, but also measure attitudes that lead to the improvement of the overall work environment.

Furthermore, scales for measuring safety attitude have been developed for various contexts, such as intensive care units and nursing homes [35, 36]. One factor measured by these scales is the teamwork environment. Cappelen cites teamwork, incident reporting and feedback, and training and skills as assessments of the state of patient safety culture [37]. These are consistent with the self-improvement for patient safety and counter measures based on incident experiences of this study.

Fourth, “nursing care to protect the lives of patients” entails viewing patient safety in the context of their lives and in partnership with the patient. As home care is performed at the patient’s home, unlike an institutional environment, patients and their family caregivers may contribute to the occurrence of adverse events. Therefore, it is necessary to consider their role when providing care [38]. Moreover, for the frail elderly to feel safe at home, it is important to have a positive approach to building relationships with the caregiver and have capable supporting staff [39]. Additionally, HVNs must respect the patients’ wishes, consider risks in their lives, and ensure patient safety in their nursing care.

### Examination of the reliability and validity of the scale

A scale measuring HVNs’ attitudes toward patient safety was evaluated for reliability and validity by scale development, EFA, CFA, and criterion-related validity. In the item analysis, EFA extracted a 4-factor 19-item scale from a 35-item pool. The fact that Cronbach’s α coefficient was 0.7 or more for all factors [16] suggests that this was appropriate. Although this scale had a TLI value of 0.886, slightly lower than the reference value > 0.9 [16], the other values met the goodness-of-fit criterion. Criterion-related validity was verified by the correlation between the scale and existing measurements. The scale was correlated with the workplace safety climate measure and the revised ethical behavior scale. Given that HVNs provide care at the patient’s home, any potential mistakes are difficult for others to detect. Education on basic ethical values is important to raise nurses’ awareness of public disclosure of malpractice by patients [40]. Nurses’ ethics influence their perceptions and behavior toward patient safety. The developed scale positively correlated with nurses’ risk aversion and good care ethics. Further, improving the safety culture is important for strengthening patient safety [41]. The scale also positively correlated with the workplace safety culture scale. From the results of EFA, CFA, and criterion-related validity, we believe that this scale has reliability and validity. Thus, the developed scale is considered capable of measuring HVNs’ attitudes toward patient safety.

### Limitations

In this study, EFA was conducted, and the reliability and validity of a scale measuring HVNs’ attitudes toward patient safety were verified. The response rate of this survey was low (22%), which may affect the generalizability of the study. This is not surprising as previous studies on nurses’ patient safety perceptions also tended to have a low response rate of approximately 30% [3, 31]. This is probably caused by nurses’ difficulty in committing to the questionnaire survey due to their heavy workload. For example, in Japan, HVNs are responsible for making health observations and visiting COVID-19 patients receiving home care [42]. Relatedly, the HVNs who participated in this study may have been considerably interested in patient safety, which could have affected the results. In addition, while the CFA confirmed the scale’s compatibility, the value was slightly low. The accuracy of the scale needs to be improved to more effectively measure HVNs’ attitudes toward patient safety. To do so, additional research with more participants is recommended.

## Conclusion

In this study, we created a scale measuring HVNs’ attitudes toward patient safety based on the characteristics of home-visiting nursing practice. The reliability and validity of the scale, which comprises 19 items across 4 factors, was verified. The items included “nursing care to protect the lives of patients,” which represents HVNs’ characteristic of practicing nursing care in the patient’s home. Additionally, they included “self-improvement for patient safety,” “incident awareness,” and “counter measures based on incident experience,” which are both the nurses’ and agencies’ efforts to ensure patient safety. In other words, the scale covers the nurses’ behavior regarding (actions to ensure) patient safety and accident prevention and improvement of their awareness. Therefore, we consider that this scale can measure HVNs’ behavior, awareness, and attitudes toward patient safety. By using this scale, it is assumed that HVNs’ behavior and awareness of patient safety could be improved and that a safe life for patients could be promoted.

## Data Availability

The datasets supporting the conclusions of this article are included within the article.

## List of Abbreviations

AVE: average variance extracted
CFA: confirmatory factor analysis
CFI: comparative fit index
CR: composite reliability
CVI: content validity index
EFA: exploratory factor analysis
FTE: full-time equivalent
HVN: home-visiting nurse
I-T correlations: item-total correlations
KMO: Kaiser-Meyer-Olkin
RMSEA: root mean square error of approximation
TLI: Tucker-Lewis Index
